# Cervical human papillomavirus prevalence, risk factors and outcomes in a cohort of HIV-infected women in Harare, Zimbabwe

**DOI:** 10.4102/sajhivmed.v21i1.1123

**Published:** 2020-11-05

**Authors:** Ardele M. Mandiriri, Margaret J. Pascoe, Tinei Shamu, Sara Lowe

**Affiliations:** 1 Newlands Clinic, Harare, Zimbabwe; 2 Department of Epidemiology, Faculty of Epidemiology and Population Health, London School of Hygiene and Tropical Medicine, London, United Kingdom; 3 Institute of Social and Preventive Medicine, University of Bern, Bern, Switzerland; 4 Department of Medicine, College of Health Sciences, University of Zimbabwe, Harare, Zimbabwe

**Keywords:** cervical cancer screening, HPV infection in WLHIV, hrHPV prevalence, visual inspection with acetic acid, HPV vaccine cross protection

## Abstract

**Background:**

Human papillomavirus (HPV) associated invasive cervical cancer (ICC) is common in Zimbabwe, disproportionately affecting women living with HIV (WLHIV). Understanding high-risk HPV (hrHPV) infection in relation to cervical disease is important for ICC prevention amongst WLHIV.

**Objectives:**

To describe the prevalence of cervical hrHPV, type distribution, associated risk factors and ICC screening outcomes in an urban cohort of Zimbabwean women.

**Methods:**

In this cohort study, WLHIV were tested for hrHPV infection using the Cepheid Xpert® HPV assay and followed up for incident cervical disease. Follow-up assessments were done by visual inspection with acetic acid (VIA). Descriptive statistics and logistic regression were used to describe hrHPV burden and association between hrHPV and potential risk factors. Incidence rates (IR) and rate ratios of cervical disease by hrHPV infection status were also calculated.

**Results:**

Amongst 321 WLHIV recruited, hrHPV prevalence was 24.9% (*n* = 80). Fifty-two of these women (65%) were positive for hrHPV types other than 16 or 18/45. Younger age (22–29 years), early sexual debut (13–16 years) and antiretroviral therapy (ART) regimen (second-line ART) were independently associated with hrHPV positivity. Positive VIA IR ratio between hrHPV-positive and -negative women was 12.57 (95% confidence interval [CI]: 4.14–38.19). Only women with hrHPV infection had incident cervical disease (IR: 6.41/100 person-years, (95% CI: 3.33–12.32). There were no ICC cases by the end of the 2-year follow-up.

**Conclusion:**

There was a high prevalence of hrHPV infection other than 16 and 18/45 in this cohort. Integrating HPV testing in cervical cancer screening programmes may increase screening intervals in hrHPV-negative women, reducing costs for programmes. We recommend further research into cross protectivity of the bivalent and quadrivalent HPV vaccines against these other hrHPV types.

## Introduction

Zimbabwe is one of the countries with the highest human immunodeficiency virus (HIV) burden in sub-Saharan Africa. In 2018, the HIV prevalence was estimated at 12.7% amongst adults aged 15 to 49 years, with a higher prevalence in women (15.4%) than in men (10.0%).^1^ Women living with HIV (WLHIV) have higher rates of infection with high-risk Human Papillomavirus (hrHPV) genotypes. High-risk human papillomavirus co-infection with HIV is associated with decreased clearance, increased HPV persistence and progression to precancerous lesions and cervical cancer.^2,3,4,5^ Invasive cervical cancer (ICC) is the most common female malignancy in Zimbabwe and is the leading cause of female cancer-related deaths with an age-standardised mortality rate of 46 per 100 000 women per year.^6^

The current recommended cervical cancer screening method in low resource settings such as sub-Saharan Africa is visual inspection of the cervix after application of acetic acid (VIA).^7,8,9^ The WHO 2013 cervical cancer guidelines recommend primary HPV testing as the preferred method where resources are available, as HPV testing is more sensitive and effective in identifying women at high risk for precancerous and cancerous lesions.^10,11^ This modality of screening has not been rolled out in the region because of the prohibitive cost of the test. The diagnostic landscape is changing, and as cost-effective point-of-care HPV DNA tests are becoming more accessible, countries have commenced HPV screening as the preferred primary cervical cancer screening method.^12,13^

The Zimbabwean government partnering with GAVI, the Vaccine Alliance in 2018, rolled out a national HPV vaccination programme, as its cervical cancer primary prevention method. The programme targeted the countries’ 800 000 girls aged 10–14 using the bivalent vaccine, which confers protection to hrHPV subtypes 16/18/45.^14^ This, however, has raised questions on the cross protective effect of other hrHPV subtypes, which have been reported to be prevalent amongst women in Zimbabwe.^15,16^

Sub-Saharan African studies investigating HPV prevalence in WLHIV have shown a wide variability across different countries from 24% to 64%.^17,18,19,20,21,22^ In 2017, a Zimbabwean study showed a hrHPV prevalence of 33% in a rural cohort of 123 WLHIV. Types 35, 52 and 58 were found to be amongst the highly prevalent types, in addition to types 16 and 18.^15^ These geographical differences in HPV burden and type distribution highlight the need to assess the Zimbabwean urban context independently. In this cohort study, we investigated the prevalence of hrHPV, the associated risk factors and VIA outcomes during 2 years of follow-up.

## Methods

### Study design and setting

This analytic cohort study was conducted amongst WLHIV at Newlands Clinic (NC), in Harare, Zimbabwe. The NC was established in 2004 in partnership with the Ministry of Health and Child Care (MoHCC) and provides comprehensive HIV care to approximately 6500 men, women and children.^23^ Sexual reproductive health services form an integral component of HIV care at NC and include cervical cancer screening, diagnosis and treatment of sexually transmitted infections (STIs), and the provision of short- and long-acting reversible contraceptive methods. An HPV vaccination programme was also introduced at the clinic in 2015. In this year alone, 517 adolescents and youth 12–22 years old (312 females and 205 males) were vaccinated using the quadrivalent HPV vaccine. The vaccination of children, adolescents and young adults still continues to date at the clinic as part of the clinic’s effort to prevent a potential burden of HPV-related cancers in these young people as they mature.

Women registered in care at NC attend routine annual cervical cancer screening where the VIA method is used as guided by the Zimbabwean Ministry of Health. The procedure is performed by a team of three nurses who have been trained in this technique. Women with a positive VIA screen are treated with either cryotherapy, loop electrical excision procedure (LEEP) or referred for specialist management depending on the type and extent of the cervical lesion. Visual inspection with acetic acid and cryotherapy are conducted by the nurses. The LEEP procedure is performed by a gynaecologist, and the excised tissue is sent to an external laboratory for histological examination.

### Study procedure

We invited women attending cervical cancer screening between 01 September 2017 and 21 November 2018 to participate in the study. Sexually active, HIV-positive women above the age of 18 years who consented to participate in the study were recruited. Potential participants with a positive pregnancy test were excluded. Baseline information including sexual history and demographic information were collected and stored in a Microsoft Access database.

At the baseline visit, we screened participants using VIA, the study nurses collected endocervical swabs for the HPV DNA tests. Women who screened positive by VIA were reviewed six monthly, and those who screened negative, annually. For the HPV DNA tests, we collected endocervical swabs using the Cervexbrush25 and deposited the samples into Preservcyt50 transport medium. We used Xpert® HPV kits for testing on the GeneXpert machine located at the onsite NC laboratory. The test detected 14 high-risk HPV with callouts for HPV 16, HPV 18/45 and ‘other hrHPV’. Amongst the ‘other hrHPV’ subtypes are HPV 31, 33, 35, 39, 51, 52, 56, 58, 59, 66 and 68. The 14 HPV types are detected in five fluorescent channels, each with individual parameters for target detection and validity; channel 1: HPV16, channel 2: HPV18/45, channel 3: HPV31/33/35/52/58, channel 4: HPV51/59, channel 5: HPV 39/56/66/68. For channels in which more than one type is detected, the Xpert^®^ HPV test does not distinguish between types.^24^ Positive hrHPV results during the study period did not alter VIA follow-up intervals which were determined by VIA results only. We followed up participants with VIA screening for a median of 26 months (interquartile range [IQR]: 24–28), which was up to April 2020. Participants with incident HPV-related disease as high-grade vulva intraepithelial neoplasia (VIN3) were censored from further follow-up.

We collected additional patient data including antiretroviral therapy (ART) medication history, VIA screening results and laboratory results from the clinic’s electronic database. None of the women in the study cohort had an HPV vaccination history.

### Statistical analysis

We used Stata 13.1 (College Station, Texas) for data cleaning and analysis. We used proportions and medians for descriptive statistics, logistic regression and the associated Wald test for measures of association between hrHPV test and potential risk factors, and Wilcoxon Rank-Sum (Mann Whitney) test differences between continuous variables. We assessed all potential risk factors in univariate and multivariable logistic regression models to determine independent associations. Our final logistic regression model did not include VIA data as HPV infection is known to precede VIA status. Incidence rates (IR) and IR ratios for cervical disease defined as VIA positivity, high-grade squamous intraepithelial neoplasia (HGSIL) and cervical cancer were assessed with the follow-up data. All statistical tests were two-tailed with *p* < 0.05 considered significant and 95% confidence intervals [CI] used. In summary, our descriptive, univariate and multivariate analysis were based on all data 100% (*n* = 321). Follow-up data was based on 96% (*n* = 308) women with hrHPV test results and without incident cervical disease.

### Ethical consideration

The study was approved by the Newlands Clinic Research team and the Medical Research Council of Zimbabwe (Approval MRCZ/A/1980).

## Results

We recruited 321 women with a median age of 44 years (IQR: 38–50). One hundred and thirty-eight (43.0%) women were married and 29.6% (*n* = 95) were widowed. Two-thirds of the women (63.9%, *n* = 205) were educated to high-school level (at least 8 years of formal education). Most women (64.5%, *n* = 207) reported sexual debut between 17 and 21 years, and 13.4% (*n* = 43) were 16 years or below at sexual debut. Regarding duration on ART, 233 (70.8%) had been on ART for more than 5 years (median ART duration = 7.8 years, IQR: 4.4–10.8 years). In total, 252 (78.5%) were on a first-line ART regimen (1 Non-nucleoside Reverse Transcriptase Inhibitor [NNRTI] + 2 Nucleoside/Nucleotide Reverse Transcriptase Inhibitor [NRTI]) and had a median CD4 count of 525 cells/mm^3^ (IQR 366–687 cells/mm^3^. One-third (33.7%, *n* = 108) reported that they were unaware of their partner’s HIV status and 15.6% (*n* = 50) were in HIV sero-discordant relationships. Amongst the 310 women who had HIV viral load results, 91.2% (*n* = 283) had undetectable viral loads (below 50 copies/mL) and 2.9% (*n* = 9) had a viral load above 1000 copies/mL (Table 1).

**TABLE 1:** Socio-demographic characteristics and risk factors of participants at baseline (*N* = 321).

Characteristic	Frequency (%)†					
	hrHPV negative *N* = 241		hrHPV positive *N* = 80		Overall *N* = 321	
	*n*	%	*n*	%	*n*	%
**Age (years), median (IQR)**	44	39–50	42	37–49	44	38–50
**Age bands (years)**						
22–29	11	4.6	10	12.5	21	6.5
30–45	121	50.2	36	45.0	157	48.9
46–60	89	36.9	32	40.0	121	37.7
> 60	20	8.3	2	2.5	22	6.9
**Marital status**						
Divorced	23	9.5	14	17.5	37	11.5
Married	107	44.4	31	38.8	138	43.0
Separated	10	4.1	3	3.8	13	4.1
Single	31	12.9	7	8.8	38	11.8
Widowed	70	29.1	25	31.3	95	29.6
**Gravidity**						
0	7	2.9	4	5.0	11	3.4
1–3	144	59.8	47	58.8	191	59.5
> 3	90	37.3	29	36.3	119	37.1
**Contraception**						
Hormonal	64	26.6	29	36.3	93	29.0
Non-hormonal	47	19.5	20	25.0	67	20.9
None	130	53.9	31	38.8	161	50.2
**Education**						
None	12	5.0	6	7.5	18	5.6
Primary school	39	16.2	13	16.3	52	16.2
High school	153	63.5	52	65.0	205	63.9
Tertiary education	37	15.4	9	11.3	46	14.3
**Age of sexual debut**						
< 13	3	1.2	2	15.0	5	1.6
13–16	23	9.5	15	63.8	38	11.8
17–21	156	64.7	51	18.8	207	64.5
> 21	59	24.5	12	2.5	71	22.2
**Partner HIV status**						
Negative	40	16.6	10	12.5	50	15.6
Positive	123	51.0	40	50.0	163	50.8
Unknown	78	32.4	30	37.5	108	33.6
**VIA‡: At HPV screening**						
Positive	3	1.2	6	7.5	9	2.8
Negative	237	98.3	74	92.5	311	96.9
Missing	1	0.4	-	-	1	0.3
**Historical VIA outcomes 2 years prior to HPV sample**						
All negative	226	93.8	57	28.8	283	11.8
Any positive	15	6.2	23	71.3	38	88.1
**Historical STI’s 2 years prior to HPV sample**						
Any STI	202	16.2	59	26.2	261	18.7
Non-Viral STI	39	83.8	21	73.8	60	81.3
**Duration of ART (years), median (IQR)**	7.6	4.6–10.5	8.0	3.5–11.0	7.8	4.4–10.8
**Viral load status§ (copies/mL)**						
< 50	218	90.5	65	81.3	283	3.4
50–199	10	4.2	5	6.3	15	2.8
200–1000	0	0.0	3	3.8	3	0.9
> 1000	6	2.5	3	3.8	9	4.8
Missing	7	2.9	4	5.0	11	88.2
**CD4 count (cells/mm^3^)**						
Median (IQR)	515	360–685	481	354–622	513	354–677
< 200	10	4.2	4	5.0	14	4.4
≥ 200	231	95.9	76	95.0	307	95.6
**ART regimen**						
ART naïve	4	1.7	0	0.0	4	1.3
First line	200	83.0	56	70.0	256	81.0
Second line	36	14.9	24	30.0	60	18.7
Third line	1	0.4	0	0.0	1	0.3

VIA, visual inspection with acetic acid; ART, antiretroviral therapy; IQR, interquartile range; hrHPV, high-risk HPV; HPV, Human papillomavirus; STI, sexually transmitted infection.

† , Frequency unless otherwise stated.

‡ , One patient had cytology instead of VIA at HPV screen.

§ , Eleven patients did not have viral load.

**FIGURE 1:**
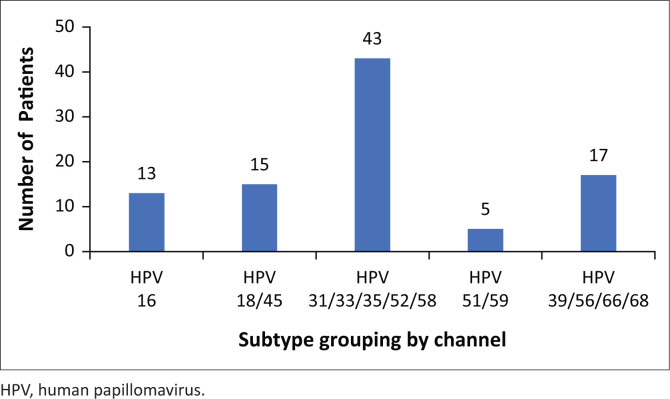
Distribution of high-risk human papillomavirus infection in women living with human immunodeficiency virus (*n* = 80) as determined by five channel Xpert^®^ human papillomavirus.

All the 321 women had a successful HPV test, 80 (24.9%; 95% CI: 20.2% – 29.7%) were positive for any hrHPV. Human papillomavirus 16 positivity without other coinfections was in 9 (11.2%) women, 10 (12.5%) were positive for HPV 18/45 alone, 52 (65%) for *other* hrHPV. A small proportion, four (5%) had HPV 16 and *other* hrHPV coinfections, and five (6.3%) had HPV 18 and *other* hrHPV. There were no co-infections with HPV 16 and HPV 18/45. Data for specific channels positive for *other* hrHPV-positive samples were available for 56/61 samples (91.8%). Amongst these 56 samples, 40 (71.4%) were positive in channel 3 (HPV31/33/35/52/58), 5 (7.1%) in channel 4 (HPV51/59) and 15 (26.8%) in channel 5 (HPV 39/56/66/68). One was positive in both channels 3 and 4, whilst two were positive in both channels 3 and 5 (Figure 1). Of the 80 women with a positive hrHPV test, 6 (7.5%) had a concurrent VIA positive result. Of these women, two were positive for HPV type 16 only, one for HPV 16 and other types (channel 5) and three tested positive for other hrHPV types (2 were channel 3 positive and the other had missing channel data).

In the univariate analysis shown in Table 2, women aged 22–29 years (OR 3.0, 95% CI: 1.2–7.9, *p* = 0.02), women with a positive VIA within 2 years prior to HPV screening (OR 6.1, 95% CI: 2.9–12.8, *p* < 0.001), women on second-line ART (OR 2.38, 95% CI: 1.3–4.4) and those with a positive VIA at time of HPV screen (OR 6.6, 95% CI: 1.6–27.6, *p* < 0.001) were more likely to test positive for hrHPV. Having a detectable viral load at the time of screening and sexual debut between the age of 13 and 16 years were also significantly associated with hrHPV positivity (OR: 2.3, 95% CI: 1.01–5.3, *p* = 0.04, OR:3.2, CI: 1.3–7.9, *p* < 0.01). However, no statistical significance for hrHPV positivity was observed for CD4 count < 200 cells/mm^3^ (OR 1.2, CI: 0.4–4.0). In the multivariate analysis, only younger age (22–29 years), (adjusted odds ratio [aOR] 2.9; 95% CI: 1.0–8.8, *p* = 0.05), ART regimen (aOR 2.1; 95% CI: 1.1–4.0, *p* < 0.05) and early sexual debut at ages 13–16 years (aOR 4.0, 95% CI: 1.4–11.5, *p* < 0.05) showed evidence of association with hrHPV positivity (Table 2).

**TABLE 2:** Univariate and multivariate analysis of patient characteristics associated with high-risk human papillomavirus positivity.

Characteristic	OR (95% CI)	p**	aOR (95% CI)	p**
**Age bands (years)**				
22–29	3.0 (1.2–7.9)	0.02	2.9 (1.0–8.8)	0.05
30–45	-	-	-	-
46–60	1.2 (0.7–2.1)	0.50	1.6 (0.8–3.2)	0.17
> 60	0.3 (0.1–1.5)	0.15	0.3 (0.1–1.7)	0.18
**Marital status**				
Married	0.8 (0.5–1.3)	0.38	0.8 (0.4–1.5)	0.49
Single	-	-	-	-
**Contraception**				
Non-hormonal	-	-	-	-
Hormonal	1.6 (0.9–2.7)	0.10	1.3 (0.7–2.6)	0.42
**Gravidity**				
0	-	-	-	-
1–3	0.6 (0.2–2.0)	0.39	0.7 (0.1–3.2)	0.62
> 3	0.6 (0.2–2.1)	0.39	0.7 (0.1–3.7)	0.67
**Education**				
None	2.1 (0.6–7.0)	0.25	2.1 (0.5–8.4)	0.29
Primary school	1.4 (0.5–3.6)	0.52	0.8 (0.3–2.4)	0.66
High school	1.4 (0.6–3.1)	0.41	1.0 (0.4–2.3)	0.98
Tertiary	-	-	-	-
**Age of sexual debut**				
< 13	3.3 (0.5–21.8)	0.22	4.6 (0.6–33.8)	0.13
13–16	3.2 (1.3–7.9)	0.01**	4.0 (1.4–11.5)	0.01**
17–21	1.6 (0.8–3.2)	0.18	1.4 (0.6–3.0)	0.41
> 21	-	-	-	-
**VIA at HPV sample collection**				
Positive	6.6 (1.6–27.6)	0.001	-	-
Negative	-	-	-	-
**Viral load (copies/mL)**				
< 50	-	-	-	-
≥ 50	2.3 (1.0–5.3)	0.04	1.6 (0.6–4.2)	0.34
**STI history in the previous 2 years**				
Any STI	-	-	-	-
Non-viral STI	2.0 (1.0–3.4)	0.05	1.95 (1.0–3.9)	0.06
**CD4 count (cells/mm^3^)**				
< 200	1.2 (0.4–4.0)	0.75	0.82 (0.2–3.2)	0.77
≥ 200	-	-	-	-
**VIA positive history in previous 2 years**				
Negative	-	-	-	-
Positive	6.1 (2.9–12.8)	< 0.001	-	-
ART regimen				
First-line ART	-	-	-	-
Second-line ART	2.38 (1.3–4.4)	0.003**	2.1 (1.1–4.0)	0.03**

VIA, visual inspection with acetic acid; ART, antiretroviral therapy; hrHPV, high risk HPV; HPV, Human papillomavirus; STI, sexually transmitted infection; CI, confidence interval; OR, odds ratio; aOR, adjusted odds ratio.

** , Wald test *p*-values for variables associated with HPV infection in the univariate and bivariate analysis.

### Visual inspection with acetic acid follow-up outcomes after high-risk human papillomavirus testing

**FIGURE 2:**
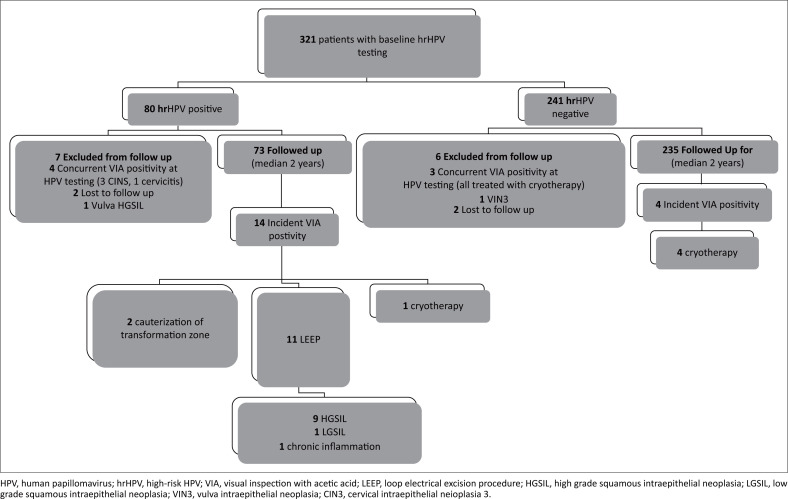
Visual inspection with acetic acid follow-up outcomes after baseline high-risk human papillomavirus testing.

Three hundred and eight (96%) participants with negative VIA at the time of hrHPV screen were followed up for a median of 2 years (645 total person-years of follow-up). Follow-up was made to determine incident VIA positivity, incident HGSIL and incident cervical cancer (confirmed by LEEP biopsy) by HPV infection status at baseline. The VIA positivity IR in hrHPV-negative women was 0.79 per 100 person-years (95% CI: 0.30–2.11). In hrHPV-positive women, the VIA positivity IR was 9.97 per 100 person-years (95% CI: 5.90–16.83). Incident rate ratio between the two groups of women was 12.57 (95% CI: 4.14–38.19). There was no evidence of incident cervical disease (HGSIL) in women who tested negative for hrHPV infection. Amongst women with hrHPV infection, the IR of HGSIL was 6.41 per 100 person-years (95% CI: 3.33–12.32). During the follow-up period, there was no incident of cervical cancer regardless of hrHPV infection status (Figure 2).

Of the 14 incident VIA-positive women at follow-up, eight (57%) at baseline were positive for other hrHPV subtypes (four being channel 3 subtypes and the others having missing channel data), two were positive for HPV16, one was positive for HPV 16 and other hrHPV subtypes (channel 5). Two were positive for hrHPV 18/45 only and one for hrHPV 18/45 and other (channel 3). None of the women had 16/18/45 coinfections at baseline. None of the women with positive channel 4 subtypes had incident cervical disease at follow-up.

## Discussion

We detected hrHPV infection in 24.5% of WLHIV attending routine cervical screening. The prevalence of types 16 and 18/45 was relatively low, with a predominance (65%) of the non-16/18/45 HPV subtypes. Our observed overall hrHPV prevalence is similar to the 33% prevalence reported in a cohort of WLHIV in rural Zimbabwe,^15^ but lower than that observed in other regional studies from Malawi, Burundi, Kenya and South Africa, which range from 45% to 64%.^19,20,25,26^ The difference in reported prevalence rates between studies may be because of several factors including the heterogeneity of the study populations. Our study describes prevalence in an urban cohort of women with well-controlled HIV (95% VL < 1000 copies/mL). The majority of the women were receiving their first-line ART regimen for a median of 7 years and had previous cervical cancer screening.

Globally, HPV types 16 and 18 predominate and are responsible for most anogenital HPV-related cancers in women.^27^ In our study, 35% of women with hrHPV had either HPV 16 or 18/45, whilst 65% had ‘other’ high-risk types. These findings are consistent with data from other studies in the region, including a world regional HPV survey, which describes an increased probability amongst WLHIV to harbour other hrHPV types such as 31, 33, 35, 52 and 58, often in the absence of cervical disease (HGSIL or ICC).^22,28,29,30^. Cumulative risk and annual rate of progression to cancer varies depending on HPV type in immunocompetent women.^31^ In a longitudinal study of 11 573 HIV-negative, hrHPV-positive women, Demarco et al. reported that 21.5% of women at Kaiser Permanente in Northern California, United States of America, with baseline HPV 16 had progressed to precancer/cancer at 7 years and women with HPV 33 also showing high cumulative risk with 18.4% progression at 7 years.^31^ Our study’s limited follow-up time inhibits us from making comments on the progression potential of the observed non-16/18/45 hrHPV subtypes. WLHIV with hrHPV are, however, at an increased risk of rapid progression to cervical disease.^2,3,4,5^ More robust epidemiological data in WLHIV are required to clearly define this risk according to hrHPV type with longer follow-up periods, in order to inform and optimise cervical screening programmes in WLHIV.

Human papillomavirus vaccination programmes are a key component of primary prevention to reduce cervical cancer-associated morbidity and mortality. The WHO currently recommends both bivalent and quadrivalent vaccines, which confer protection against hrHPV types 16 and 18.^32^ Cross protection from the bivalent vaccine for other non-vaccine high-risk types as in our cohort has been demonstrated. Van De Weele et al. noted significant reductions in incident and/or persistence of certain types (HPV 31, 33, 35 and 45), following exposure to the bivalent vaccine in a cohort of young Dutch women.^33^ In view of the dominance of subtypes other than HPV 16 or HPV 18/45, it is unclear whether the bivalent vaccine currently being administered in the Zimbabwe national programme will provide cross protection against these subtypes. An understanding of the degree of cross protection from context-specific data is warranted. This can be achieved through country-specific vaccine evaluation studies in the vaccinated cohort.

Early sexual debut and younger age were significant predictors of hrHPV infection in our cohort. Women with an early sexual debut (13–16 years) were at least three times more likely to test positive for hrHPV when compared with those whose sexual debut was after 21 years. In keeping with these findings, a cohort analysis of 1445 urban women in South Africa showed a peak in hrHPV prevalence in women younger than 25 years and a gradual decrease of hrHPV infection with increasing age of women.^34^ Van Aardt et al. postulate that the higher hrHPV prevalence in younger women may be partially explained by the lack of natural immunity in the initial stages of sexual activity.^35^ Literature also provides evidence that over 35% of women contract HPV within the first 2 years of sexual debut^8^ and that early sexual debut is also associated with a greater number of sexual partners and an increased risk of STI infection.^36,37^ Research to further elucidate clearance and progression to cervical dysplasia in younger women is also necessary.

Despite most women in our study being on first-line ART and 95% having Viral load (VL) < 1000 copies/mL, women on second-line therapy were twice as likely to test positive for hrHPV. In a systematic review of the association between HPV infection and ART, Menon et al. indicate that severe immuno-suppression of CD4 counts < 200 cells/mm^3^ increases the risk of hrHPV infection.^38^ In our context, women on second-line ART often have a history of severe immunosuppression necessitating the switch to second-line ART.

After a median of 2 years follow-up, the incident rates for both VIA positivity and HGSIL were significantly higher in the women with hrHPV infection. No cases of HGSIL were observed in hrHPV-negative women. These data are consistent with findings from large longitudinal studies. Data from the Kaiser Portland cohort of over 20 000 women and more than 15 years of follow-up demonstrate a low risk of precancer or cancer in HIV-negative women with a single negative hrHPV result. The high negative predictive value of HPV DNA testing is now well established, hence the adoption by many global cervical screening programmes as the primary screening tool.^10,39^ In low-income countries, less-frequent cervical cancer screening, once every 3 years, for hrHPV-negative WLHIV will have both the potential to reduce costs and divert scarce resources to hrHPV-positive women who are at higher risk of developing cervical cancer and in women who have never been screened. This resource optimisation may translate to greater screening potential in settings where coverage is still low like in Zimbabwe.^8^ However, a key component of the potential success of this screening programme that utilises HPV testing is the development and availability of low-cost HPV assays.

As reported in the methodology, the use of the Xpert^®^ HPV kits limited our ability to describe individual hrHPV types in the cohort apart from HPV 16. Secondly, the duration of follow-up was short and because of the nature of the pathogenesis of HPV infection, there was limited time for disease progression to occur. Thirdly, hrHPV infection was only assessed at baseline, therefore questions of HPV persistence and clearance could not be addressed. Despite these limitations, the information from this study can be used to provide background data for prospective cohort studies to ascertain persistence of HPV infections in women with controlled HIV infection.

## Conclusion

Our study highlights key epidemiological information on hrHPV infection in an urban cohort of HIV-infected women in Zimbabwe. There was a lower than expected overall prevalence of hrHPV infection in the study cohort, but a higher prevalence of hrHPV subtypes other than HPV 16 or 18/45. This raises some important vaccine effectiveness questions regarding the current roll-out of the prequalified bivalent and quadrivalent HPV vaccine, particularly regarding the level of cross-protection against other hrHPV types, which are prevalent in our setting. Our follow-up data highlights the potential benefits of integrating primary HPV testing, in cervical cancer screening programmes amongst WLHIV, which may lead to reduced screening intervals in hrHPV-negative women and potentially result in cost reduction.
